# Impaired Cardiac and Skeletal Muscle Energetics Following Anthracycline Therapy for Breast Cancer

**DOI:** 10.1161/CIRCIMAGING.123.015782

**Published:** 2023-10-17

**Authors:** David T. Gamble, James Ross, Hilal Khan, Andreas Unger, Lesley Cheyne, Amelia Rudd, Fiona Saunders, Janaki Srivanasan, Sylvia Kamya, Graham Horgan, Andrew Hannah, Santosh Baliga, Carlo Gabriele Tocchetti, Gordon Urquhart, Wolfgang A. Linke, Yazan Masannat, Ahmed Mustafa, Mairi Fuller, Beatrix Elsberger, Ravi Sharma, Dana Dawson

**Affiliations:** Cardiology Research Group, Aberdeen Cardiovascular and Diabetes Centre, School of Medicine and Dentistry, University of Aberdeen, United Kingdom (D.T.G., J.R., H.K., L.C., A.R., F.S., J.S., S.K., D.D.).; Institute of Physiology II, University of Münster, Germany (A.U., W.A.L.).; Biomathematics and Statistics Scotland, Aberdeen (G.H.).; Department of Cardiology National Health Service (NHS) Grampian (A.H.), Aberdeen Royal Infirmary, Foresterhill, Scotland, United Kingdom.; Department of Trauma and Orthopaedic Surgery (S.B.), Aberdeen Royal Infirmary, Foresterhill, Scotland, United Kingdom.; Department of Oncology NHS Grampian (G.U., R.S.), Aberdeen Royal Infirmary, Foresterhill, Scotland, United Kingdom.; Department of Breast Surgery NHS Grampian (Y.M., A.M., M.F., B.E.), Aberdeen Royal Infirmary, Foresterhill, Scotland, United Kingdom.; Department of Translational Medical Sciences (DISMET), Center for Basic and Clinical Immunology Research (CISI), Interdepartmental Center of Clinical and Translational Sciences (CIRCET), Interdepartmental Hypertension Research Center (CIRIAPA), Federico II University, Naples, Italy (C.G.T.).

**Keywords:** anthracycline, breast cancer, cardiac energetics, chemotherapy, skeletal muscle

## Abstract

**Background:**

Anthracycline-related cardiac toxicity is a recognized consequence of cancer therapies. We assess resting cardiac and skeletal muscle energetics and myocyte, sarcomere, and mitochondrial integrity in patients with breast cancer receiving epirubicin.

**Methods:**

In a prospective, mechanistic, observational, longitudinal study, we investigated chemotherapy-naive patients with breast cancer receiving epirubicin versus sex- and age-matched healthy controls. Resting energetic status of cardiac and skeletal muscle (phosphocreatine/gamma ATP and inorganic phosphate [Pi]/phosphocreatine, respectively) was assessed with ^31^P-magnetic resonance spectroscopy. Cardiac function and tissue characterization (magnetic resonance imaging and 2D-echocardiography), cardiac biomarkers (serum NT-pro-BNP and high-sensitivity troponin I), and structural assessments of skeletal muscle biopsies were obtained. All study assessments were performed before and after chemotherapy.

**Results:**

Twenty-five female patients with breast cancer (median age, 53 years) received a mean epirubicin dose of 304 mg/m^2^, and 25 age/sex-matched controls were recruited. Despite comparable baseline cardiac and skeletal muscle energetics with the healthy controls, after chemotherapy, patients with breast cancer showed a reduction in cardiac phosphocreatine/gamma ATP ratio (2.0±0.7 versus 1.1±0.5; *P*=0.001) and an increase in skeletal muscle Pi/phosphocreatine ratio (0.1±0.1 versus 0.2±0.1; *P*=0.022). This occurred in the context of increases in left ventricular end-systolic and end-diastolic volumes (*P*=0.009 and *P*=0.008, respectively), T1 and T2 mapping (*P*=0.001 and *P*=0.028, respectively) but with preserved left ventricular ejection fraction, mass and global longitudinal strain, and no change in cardiac biomarkers. There was preservation of the mitochondrial copy number in skeletal muscle biopsies but a significant increase in areas of skeletal muscle degradation (*P*=0.001) in patients with breast cancer following chemotherapy. Patients with breast cancer demonstrated a reduction in skeletal muscle sarcomere number from the prechemotherapy stage compared with healthy controls (*P*=0.013).

**Conclusions:**

Contemporary doses of epirubicin for breast cancer treatment result in a significant reduction of cardiac and skeletal muscle high-energy ^31^P-metabolism alongside structural skeletal muscle changes.

**REGISTRATION:**

URL: https://www.clinicaltrials.gov; Unique identifier: NCT04467411

**CLINICAL PERSPECTIVE**

We have demonstrated impairment of high-energy phosphate metabolism in the myocardium and skeletal muscle, alongside structural changes in the skeletal muscle, in low-risk patients with breast cancer following anthracycline chemotherapy compared with healthy controls. Although their left ventricular ejection fraction was preserved and none fulfilled the current criteria for cancer-related therapy cardiac dysfunction, the significant reductions in cardiac and skeletal energetics observed here challenge the view that anthracycline-induced cardiotoxicity occurs only in a relatively small proportion of patients.

Anthracycline-related cardiac dysfunction is a recognized consequence of cancer therapies. Stages of cardiotoxicity have been defined based on reduction of left ventricular ejection fraction (LVEF) and global longitudinal strain, and a rise in cardiac biomarkers.^1^ However, subtle myocardial changes occur much earlier than a measurable decline in the LV systolic function, demonstrated by increases in parametric (T1 and T2) myocardial mapping on cardiac magnetic resonance imaging,^2,3^ which imply the presence of myocardial edema and other histopathologic abnormalities. Experimental evidence suggests that the mechanisms responsible for these abnormalities include free radical production with consequent mitochondrial dysfunction, suppression of myofilament protein synthesis, and reduction in cardiac energetics.^4,5^

Thus far, trials of cardioprotective therapies (such as angiotensin-converting enzyme inhibitors, beta-blockers, or statins) during anthracycline chemotherapy did not prevent a small but measurable reduction in left venrticular (LV) ejection fraction (EF) after extended follow-up.^6,7^ For the success of primary prevention therapies, it would be important to have the ability to detect measurable end points directly related to specific pathways of myocyte derangement, such as mitochondria-related cardiac energetics. A decrease in cardiac energetics has been shown to precede the subsequent onset of LV dysfunction in vivo in animal models of anthracycline-induced cardiomyopathy,^4^ but it remains unknown if this is found in human patients receiving standard chemotherapy doses. Furthermore, despite the systemic toxicity profile of anthracyclines, their effects on skeletal muscle myocytes in humans are significantly under-investigated. Questions remain as to whether energetic or structural changes also occur in skeletal muscle, to indicate skeletal muscle stress.^8^ This is important as physical exercise training during chemotherapy has been proposed as a primary prevention strategy for functional disability.^9^ In this study, we assess resting cardiac and skeletal muscle energetics in patients with breast cancer undergoing anthracycline-based chemotherapy. Using skeletal muscle biopsies, we simultaneously investigate basic sarcomere and mitochondrial integrity to distinguish between functional and structural causes of anthracycline-induced toxicity.

## METHODS

Upon reasonable request, the authors will make the data, methods used in the analysis, and materials used to conduct the research available to any researcher for purposes of reproducing the results or replicating the procedure, as long as this does not compromise patient confidentiality, within 1 year of publication.

### Study Design

This was a prospective, mechanistic, observational, longitudinal investigation enrolling a cohort of anthracycline-naive female patients with breast cancer at Aberdeen Royal Infirmary between 2021 and 2022. Patients were enrolled if they were scheduled for adjuvant or neo-adjuvant anthracycline-based chemotherapy regimen as 3 cycles of 5-fluorouracil, epirubicin, and cyclophosphamide followed by 3 cycles of docetaxel. Patients were studied at 2 time points: at baseline before the first cycle of chemotherapy and a final examination taking place within 6 weeks of their last cycle of chemotherapy but before any radiotherapy-based treatment. A group of healthy control women was recruited for comparison and studied at a single time point. Study assessments included cardiac and skeletal muscle ^31^Phosphorus-spectroscopy, cardiac magnetic resonance, transthoracic echocardiography, blood sampling, and skeletal muscle biopsy. The study was approved by the Institutional Review Board (North of Scotland Ethics Committee One), and all participants provided informed consent. Two participants did not return for follow-up.

Full investigative protocols and analyses of all methodology described below are presented in the Supplemental Material. A flow diagram of recruitment and study numbers is shown in Figure S1.

### ^31^P-Magnetic Resonance Cardiac and Skeletal Muscle Spectroscopy Acquisition and Analysis

All participants were scanned on a 3T Philips scanner (Achieva, Philips Medical Systems, Best, the Netherlands). ^31^P- Magnetic Resonance Spectroscopy was acquired using a 14-cm diameter transmit/receive ^31^P surface coil (Philips Healthcare, Best, the Netherlands).^10^ Skeletal muscle ^31^P-spectrsocopy was performed using the same coil placed over the vastus lateralis muscle. For in vivo resting cardiac energetics, the phosphocreatine/γ-ATP (γATP) ratio was determined after the γATP was corrected for blood contamination and saturation correction applied as described previously.^11^ For skeletal muscle (vastus lateralis) resting energetics, the inorganic phosphate (Pi)/phosphocreatine ratio was derived.

### Cardiac Magnetic Resonance Imaging Acquisition and Analysis

A 5-channel phased array surface coil was used to acquire an ECG-gated cardiac magnetic resonance protocol inclusive of cine imaging, T1/T2 mapping, and late gadolinium enhancement. Cardiac magnetic resonance images were analyzed in Circle cvi42 v5.16 (Circle Cardiovascular Imaging, Calgary, Canada) for deriving indexed LV volumes and mass, and EF. T1 and T2 maps were analyzed with the Philips IntelliSpace software Version 11.1 (Koninklijke Philips N.V., Amsterdam, the Netherlands). The myocardial extracellular volume fraction was calculated for the whole left ventricle. Analysis of all cardiac magnetic resonance and spectroscopy was performed on anonymized data at the end of the study and blinded to the pre/post-chemotherapy status of the participants.

### Two-Dimensional Transthoracic Echocardiography and Image Analysis

Two-dimensional and Doppler Echocardiography was performed using a Vivid E9 system with a 2.5-MHz (M5S) transducer (GE Vingmed, Horten, Norway) by a British Society of Echocardiography-accredited sonographer and image analysis performed by 2 experienced operators (D.T.G. and J.S.) on the TOMTEC-ARENA v6 (Tomtec, Unterschleissheim, Germany).

### Cardiovascular Biomarkers

NT-pro-BNP (N-terminal pro-B-type natriuretic peptide) and high-sensitivity troponin I were analyzed using an Abbott Alinity I Immunoassay System Analyser (Abbott, IL).

### Skeletal Muscle Biopsies

Skeletal muscle biopsies (10 mg) from the left vastus lateralis were obtained with a Bard Magnum disposable core tissue biopsy needle (Bard Medical, Covington, GA) using an aseptic technique, immediately snap-frozen in liquid nitrogen and placed in dry storage at −80 °C until analysis.

### Real-Time Quantitative PCR

Real-time quantitative polymerase chain reaction was performed to determine mitochondrial DNA copy number. Samples were analyzed on a Roche LightCycler 480 II (F. Hoffmann-La Roche AG, Basel, Switzerland) using LightCycler 480 (v1.5.1.62 SP3) software. Data were expressed as mitochondrial copy number per 10 ng of RNA.

### Transmission Electron Microscopy and Immunofluorescence Confocal Laser Microscopy

Transmission electron microscopy was processed as described.^12^ Areas that demonstrated breakdown of the normal cytoplasmic organelles and sarcomeric apparatus were measured from electron micrographs and demarcated as areas of degradation as previously described.^13,14^ These were calculated as percentage degradation of full skeletal muscle areas (each biopsy, 225 000 µm²).

Confocal laser microscopy imaging for quantitation of sarcomere loss was done as previously described.^15^ The number of striation signals (sarcomere bands) within a 100-µm² cell area was calculated on immunofluorescent images labeled with primary antibody TTN-Z (titin). One Z-disc signal was counted as 1 sarcomere. A total of 400 to 800 antibody-marked striation signals were counted per sample. To allow for intersample comparison, we measured the immunofluorescent signals at a sarcomere length of minimum of 1.8 µm.

### Statistical Analysis

Statistical analysis was performed using IBM SPSS statistics Version 28.0. Descriptive statistics were presented as mean±SD or number (%), unless stated otherwise. To detect a change from baseline to post-chemotherapy follow-up in patients with breast cancer of 0.5 (SD difference, 0.6) for the primary end point of phosphocreatine/γATP ratio, with β=0.8, and a 2-sided significance level of 0.05, the sample size required 12 participants for paired follow-up. Comparisons between healthy volunteers and patients with breast cancer were made using ANOVA for all response variables and logistic regression for binary categorical measures. Body mass index was included as a covariate in the ANOVA and logistic regression analysis. Comparisons between patients with breast cancer at baseline and after chemotherapy were made using paired samples *t* tests. A mixed linear model was used for all other data with study visits as a fixed effect and the unique participant identifier as a random effect. Statistical significance was set at *P*<0.05.

## RESULTS

Twenty-five female patients with breast cancer and 25 age/sex-matched controls were recruited. Participants’ baseline characteristics are presented in Table 1. The median age of the breast cancer group was 53 years (range, 32–74 years), they had a mean body mass index of 30 kg/m^2^ and minimal cardiovascular comorbidities with only 2 (8%) participants receiving renin-angiotensin inhibitor treatment for hypertension. All patients with breast cancer received 5-fluorouracil, epirubicin, cyclophosphamide, and docetaxel as their chemotherapy regimen with a cumulative epirubicin dose of 304±20 mg/m^2^. Three participants (12%) also received trastuzumab. The median follow-up time from the first date of chemotherapy to their final study visit was 145 days (range, 123–265 days). The healthy volunteer group had a median age of 51 years (range, 23–71 years), a mean body mass index of 26 kg/m^2^, and were free of any health conditions.

**Table 1.**
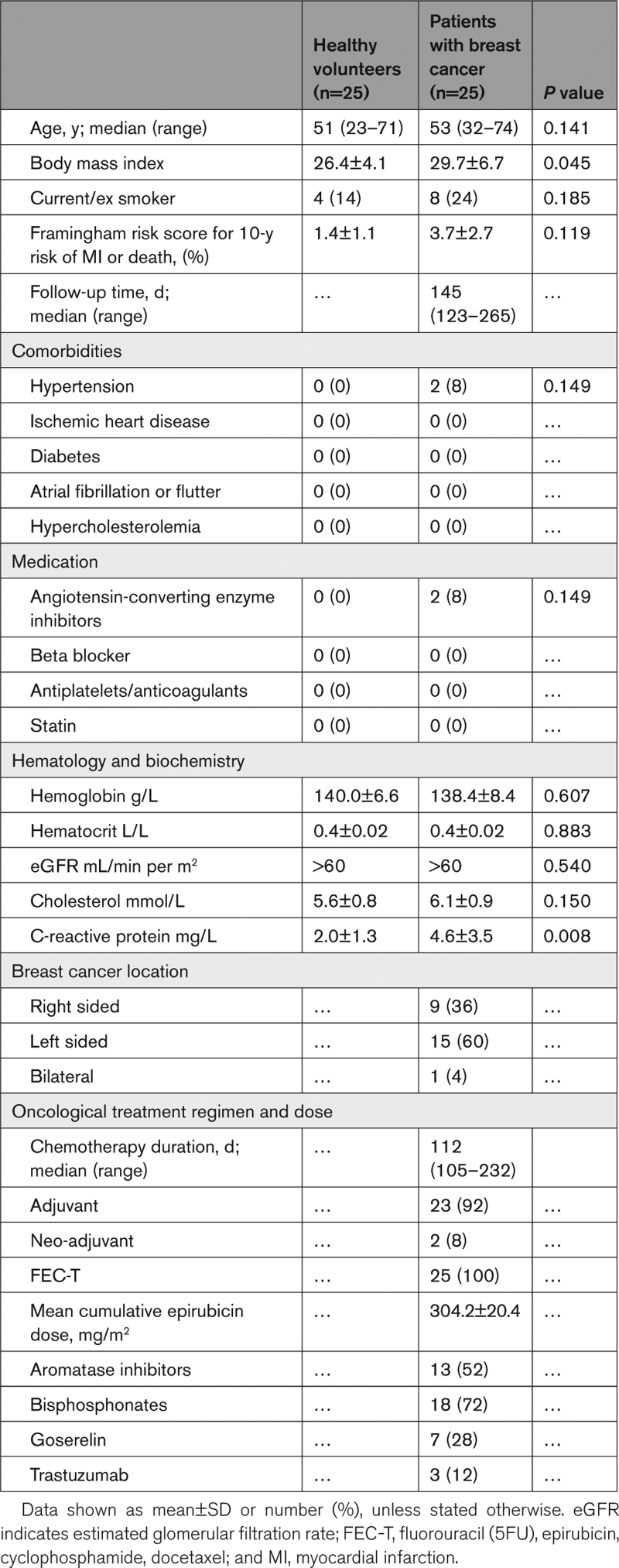
Controls and Breast Cancer Participants Clinical Characteristics, Pathology, and Treatment

### Cardiac Energetics, Cardiac Magnetic Resonance Imaging, 2D-Echocardiography, and Cardiac Biomarkers

There was no difference in the myocardial corrected phosphocreatine/γATP ratio nor the indexed LV end-diastolic or end-systolic volumes, indexed LV mass, cardiac magnetic resonance imaging, or echocardiography-derived LVEF, T1 mapping, T2 mapping or global longitudinal strain between the healthy volunteers and the patients with breast cancer before chemotherapy—Table 2.

**Table 2.**
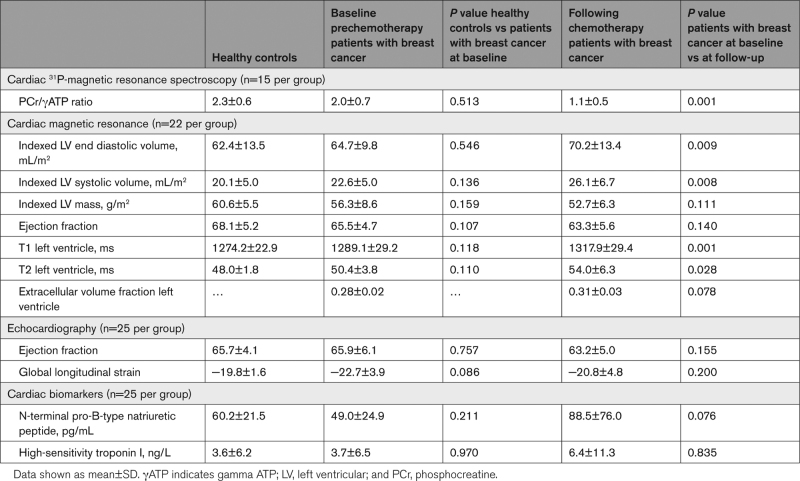
Cardiac Energetics, Cardiac Magnetic Resonance Imaging, 2D-Echocardiography, and Serum Cardiac Biomarkers

**Figure 1.**
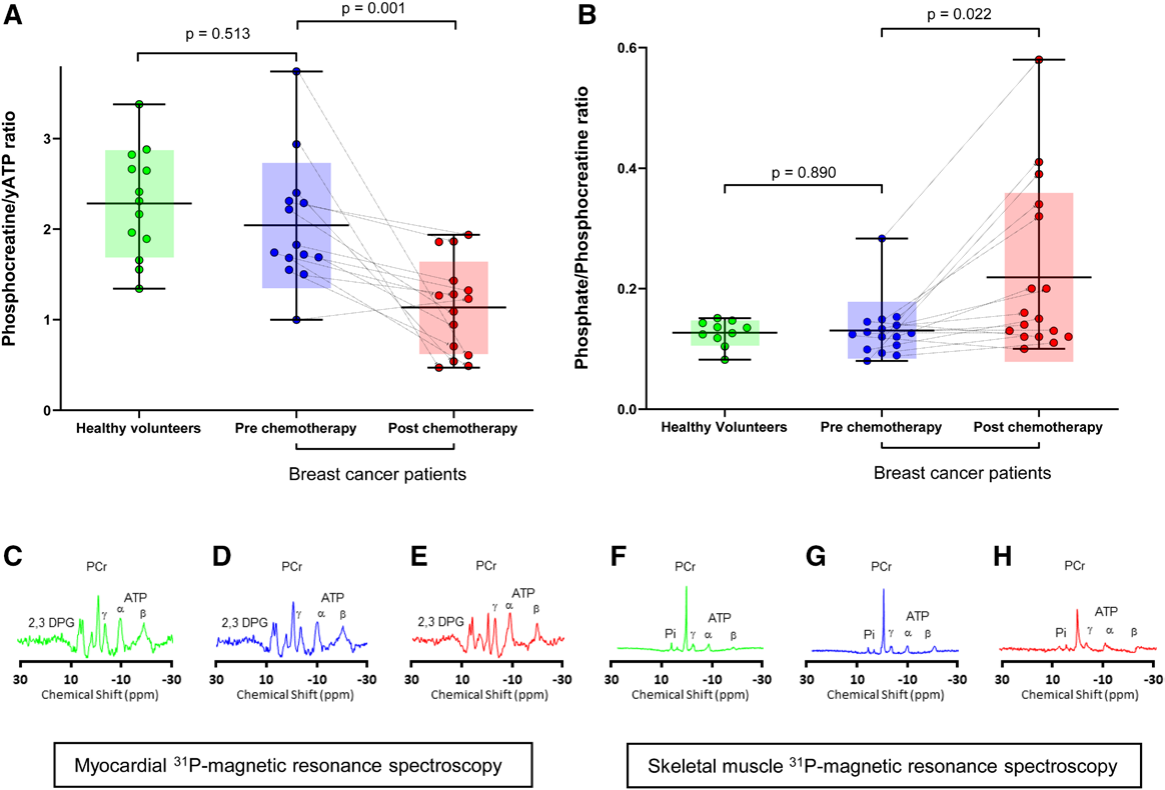
**^31^P-magnetic resonance spectroscopy for cardiac and skeletal muscle energetics.** Data shown as box plots/error bars and superimposed individual, paired data points with mean line, box for SDs and error bars for minimum and maximum. **A**, Corrected phosphocreatine (PCr)/gamma ATP (γATP) in the myocardium and (**B**) inorganic phosphate (Pi)/PCr in the skeletal muscle, for healthy controls and patients with breast cancer before chemotherapy and after chemotherapy. Below are example spectra showing PCr, γ, β, and α ATP, and 2,3-diphosphoglycerate (2,3 DPG) peaks in healthy volunteers (**C**) and in paired patients with breast cancer before (**D**) and after (**E**) chemotherapy; Pi, PCr and γ, β, and α ATP peaks in healthy volunteers (**F**) and in paired patients with breast cancer before (**G**) and after (**H**) chemotherapy.

However, following chemotherapy, patients with breast cancer had a significant reduction in the myocardial corrected phosphocreatine/γATP ratio compared with their own baseline at prechemotherapy assessment (1.1±0.5 versus 2.0±0.7; *P*=0.001)—Table 2 and Figure 1. This was accompanied by increases in indexed LV end-diastolic (*P*=0.008) and end-systolic (*P*=0.009) volumes, as well as LV native T1 (*P*=0.001) and T2 (*P*=0.028) mapping in the patients with breast cancer after chemotherapy compared with their baseline. Conversely, their LVEF, indexed LV mass, extracellular volume fraction, and echocardiography-derived global longitudinal strain remained unchanged post-chemotherapy—Table 2. Therefore, at the point of follow-up, no participants developed chemotherapy-related cardiotoxicity as defined in the European Society of Cardiology guidelines.^16^ There was no late gadolinium enhancement in patients with breast cancer before, or after chemotherapy. There was no correlation between LVEF and phosphocreatine/γATP ratio at follow-up (*P*=0.721). There was no significant difference in NT-pro-BNP (*P*=0.076) or high-sensitivity troponin I (*P*=0.835) in patients with breast cancer after chemotherapy compared with prechemotherapy or healthy controls.

### Skeletal Muscle Energetics and Structural Integrity

There was no significant difference in the Pi/phosphocreatine ratio between healthy volunteers and patients with breast cancer at prechemotherapy stage (*P*=0.890). However, there was a significant increase in Pi/phosphocreatine ratio measured in breast cancer patients’ post-chemotherapy (0.22±0.14 versus 0.13±0.05; *P*=0.022)—Table 3 and Figure 1.

**Table 3.**
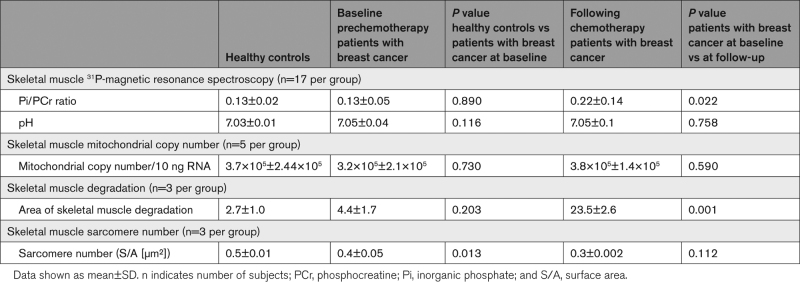
Skeletal Muscle Energetics and Structural Integrity

**Figure 2.**
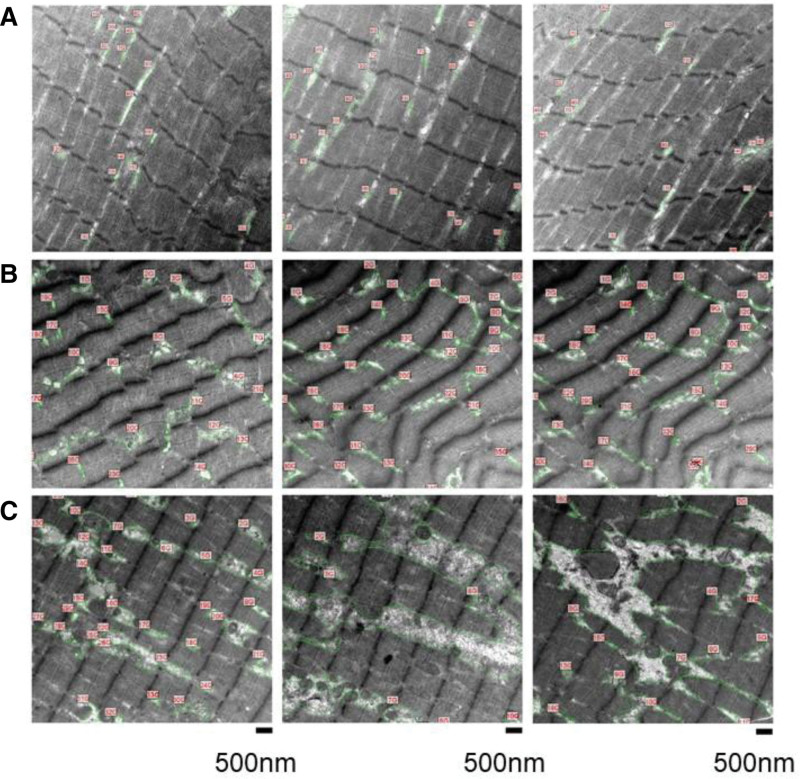
**Transmission electron microscopy of skeletal muscle showing demarcated (green contours) areas of degradation.** Examples from (**A**) healthy volunteers, (**B**) patients with breast cancer at baseline, and (**C**) patients with breast cancer following anthracycline chemotherapy.

**Figure 3.**
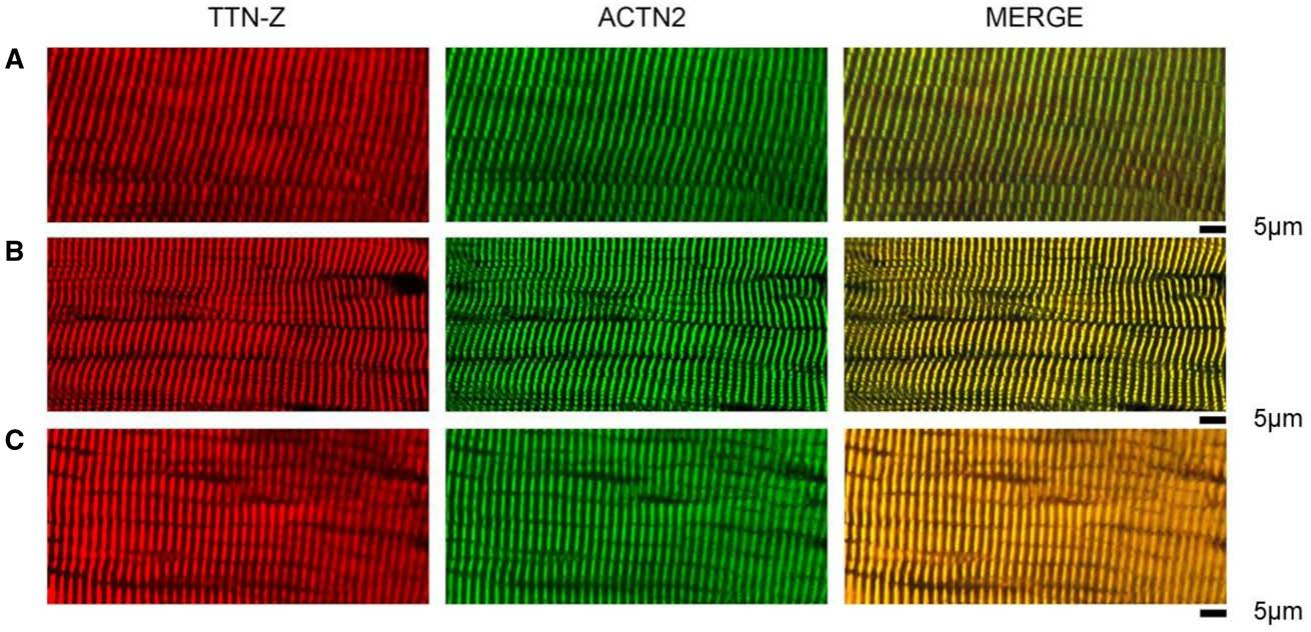
**Confocal laser scanning microscopy analysis of skeletal muscle.** Immunofluorescence staining was done against TTN-Z (titin; red) and ACTN2 (alpha-actinin-2; green) epitopes and merged (Z-disc); examples from (**A**) healthy volunteers, (**B**) patients with breast cancer at baseline, and (**C**) patients with breast cancer following anthracycline chemotherapy.

There was no significant difference in skeletal muscle pH ratio between healthy volunteers and patients with breast cancer at baseline (*P*=0.116) or between patients with breast cancer at baseline and at follow-up (*P*=0.758). Real-time quantitative phosphocreatine showed comparable copy number of mitochondria in healthy volunteers and prechemotherapy breast cancer patients (*P*=0.730). There was no loss in the number of mitochondria of skeletal muscle cells in post-chemotherapy breast cancer patients (*P*=0.590) (Table 3). Transmission electron microscopy did not show any difference in the measured area of skeletal muscle degradation between the healthy volunteers and prechemotherapy breast cancer patients (*P*=0.203). However, there was a significant increase in skeletal muscle degradation in the patients with breast cancer post- compared with prechemotherapy compared with baseline (*P*=0.001)—Table 3 and Figure 2. Immunofluorescent confocal microscopy showed a significant reduction in sarcomere count per unit area (S/A [µm²]) in patients with breast cancer compared with controls (*P*=0.013). There was no difference in sarcomere number in the patients with breast cancer before and after chemotherapy (*P*=0.112)—Table 3 and Figure 3. The observed cardiac and skeletal muscle findings remained unchanged when those participants who also received Trastuzumab (n=3) were removed from the analysis, inclusive of the primary end point of cardiac phosphocreatine/yATP ratio (1.9±0.5 versus 1.2±0.5; *P*=0.004). There was no association between participants’ body mass index and the impairments of high-energy phosphate metabolism in cardiac (*P*=0.478) and skeletal muscle (*P*=0.729).

## DISCUSSION

In this prospective study, we report significant reductions in resting cardiac and skeletal muscle energetics in patients with breast cancer after administration of standard clinical doses of anthracycline chemotherapy. There was preservation of the number of skeletal muscle mitochondria but increased skeletal muscle degradation after epirubicin-containing chemotherapy. Patients with breast cancer had reduced numbers of sarcomeres compared with healthy controls from prechemotherapy stage.

This is the first human study to demonstrate impairment of LV high-energy phosphate metabolism in patients with breast cancer following administration of contemporary doses of anthracyclines. Our center uses epirubicin, an anthracycline with a lesser cardiotoxic profile compared with the more widely used doxorubicin.^17^ The studied patient cohort had a very low cardiovascular risk profile; thus, we are confident that we have evaluated the effect of chemotherapy on myocardial energetics without the confounding contribution of other comorbidities. Despite their low cardiovascular risk profile, small increases in indexed LV volumes and parametric mapping were detected, in keeping with several previous reports.^18^ However, at this stage of follow-up, we did not observe a reduction in the LVEF nor LV mass, both of which may have been adversely affected by more prevalent comorbidities in previously reported patients.^19,20^ It is known from experimental animal models that increase in myocardial T1/T2 mapping reflective of myocardial edema, intracytoplasmic vacuolization, and myofibril loss are detectable as early as 6 weeks.^21,22^ After chemotherapy, our patients with breast cancer also showed increases in T1/T2 mapping but having not done multiple points-serial assessments, we were not able to assess the relative time points of resting cardiac energetics decline versus increased parametric mapping during chemotherapy. The main purpose of our investigation was to detect if resting myocardial energetics is adversely affected in human patients, which we herewith confirmed. Importantly, this was detected in the absence of a decline in LVEF or loss of myocardial mass or increase of cardiac biomarkers. In a recent prospective study, obesity was associated (odds ratio, 3.02 [95% CI, 1.10–8.25]; *P*=0.03) with an important increase in risk-related cardiotoxicity^23^; however, in our study, there was no association between participants’ body mass index and the impairments of high-energy phosphate metabolism in cardiac and skeletal muscle. In a recent meta-analysis, most studies demonstrated a reduction in LVEF following doxorubicin therapy.^24^ However, in our study, no participants developed chemotherapy-related cardiotoxicity as defined in the European Society of Cardiology guidelines,^16^ at the point of follow-up. This reinforces prior evidence that epirubicin may be less cardiotoxic. The heart is a known omnivore, able to generate energy from glycolysis, fatty acid oxidation, as well as, other substrates such as ketone bodies^25^ or branched-chain amino-acids,^26^ to meet the high-energy requirements of 6 kg ATP per day. Therefore, the demonstration of energetics impairment opens new avenues of exploring cardioprotection, for example, with metabolic modulators^27^ or medications that preferentially change metabolic substrate availability.^28^ It has been demonstrated that empagliflozin improves cardiac remodeling and heart failure in animal models leading to a switch in myocardial fuel utilization away from glucose with consequent improvement in myocardial energetics and LV systolic function.^29^ These findings suggest that targeting mitochondrial energetics directly may represent a new avenue for chemotherapy-induced cardiotoxicity therapeutics. While anthracycline-based chemotherapy is well known to negatively impact cardiac function, the impact on skeletal muscle has received much less attention. Our findings substantiate those demonstrated in a recent prospective cohort study in patients with breast cancer treated with anthracycline chemotherapy, which also showed an increase in pi/phosphocreatine ratio in patients after chemotherapy, both at rest and after incremental intensity workloads.^30^ Increasing pi/phosphocreatine ratio reflects greater ADP accumulation which triggers increased phosphocreatine breakdown to compensate for inadequate ATP production.^31^ Our data show that the number of skeletal muscle mitochondria was comparable between cancer patients and healthy controls, both before and after chemotherapy. The observation that mitochondrial number was not reduced in our study suggests that the energetic impairment is related to the function of mitochondria not their overall number at this early stage. We also noted other significant perturbations of muscle homeostasis. First, cancer patients had reduced skeletal muscle sarcomere number, suggesting that the well-known associated sarcopenic process previously described in late cancer stages^32,33^ might in fact commence much earlier than previously appreciated. Such reductions in skeletal muscle sarcomere number have been documented in under-stretch conditions including chronic immobilization, this change in the number of contractile units leads to reduced active force generation within muscle fibers.^34^ To our knowledge, this is the first time such findings have been reported and it may represent a systemic feature of cancer. The skeletal muscle sarcomere number in our study was not negatively affected by chemotherapy administration, although we acknowledge the sample size may have been too small to conclude on this. Second, we observed an increase in areas of muscle degradation on electron microscopy after chemotherapy administration. These likely represent disrupted cytoplasmic organelles representing muscle breakdown, which are usually the precursors of fatty infiltration and muscle fibrosis.^35,36^ These add further to the knowledge of anthracycline-based chemotherapy being causative in the loss of skeletal muscle mass resulting in sarcopenia^37^ and impaired contractile function.^38^ These degradation areas show a transition of muscle cells into lipomatous tissue and granular-filamentous aggregates seen in primary myopathies or myopathies secondary to chronic systemic conditions.^39^ Future studies should focus on correlating skeletal muscle degradation with functional assessments, to further explore their impact on functional capacity. These findings of structural changes and impaired energetics within the skeletal muscle may provide explanation for the high prevalence of fatigue and functional disability^40^ in these patients. These have direct clinical relevance as exercise rehabilitation during active chemotherapy is part of the current guidelines and recommendations and within the patients’ anticipated expectations.^9^ Perhaps unsurprisingly, the early results from randomized controlled studies of exercise training in patients with breast cancer showed that despite an increased peak oxygen consumption derived from training, functional disability was not attenuated.^41^ Further work is needed to address primary prevention of cardiac and skeletal muscle dysfunction resulting from cancer therapies.

### Limitations

This was a single-center study with a relatively small sample size of low cardiac risk patients with breast cancer. Our findings are confined to the chemotherapy regimens applied within this study. Due to the predominance of White women with breast cancer in our catchment area, our results may not be applicable to other ethnicities or higher risk patients, or those with a different malignancy or clinical profile. The relatively small number of skeletal muscle biopsy assessment in this study is a limitation and future studies in larger numbers should be conducted to confirm these findings.

### Conclusions

We demonstrate the impairment of high-energy phosphate metabolism in the myocardium and skeletal muscle of patients with breast cancer following anthracycline chemotherapy. We also demonstrated structural changes in the skeletal muscle both predating and after chemotherapy in cancer patients compared with healthy controls.

## ARTICLE INFORMATION

### Acknowledgments

The fellow (Dr Gamble) recruited participants, scheduled, coordinated, and performed all clinical imaging investigations, patients skeletal muscle biopsies and venesection, conducted mitochondrial copy number analysis of muscle biopsies under supervision, analyzed all data, performed statistical analyses under supervision, and drafted this article. H. Khan and A. Rudd helped with the investigations and reviewed and contributed to this article. S. Baliga provided the healthy volunteer skeletal muscle biopsies. Dr Ross designed and developed the protocol for cardiac and skeletal muscle spectroscopy. L. Cheyne supervised muscle biopsy analyses. Drs Unger and Linke performed the skeletal muscle transmission electron microscopy and immunofluorescence confocal microscopy investigations. Dr Horgan is the study statistician. Drs Urquhart, Masannat, Elsberger, Fuller, Mustafa, and Sharma identified and recruited participants and reviewed and contributed to this article. Drs Hannah, Sharma, and Saunders contributed to the design of the study. D. Dawson (PI) designed the study, obtained funding (together with Drs Sharma and Masannat) and regulatory approvals, supervised the unfolding of the study, its analyses and revised the article drafts.

### Sources of Funding

Tenovus Scotland G18.01, D. Dawson and Dr Sharma, Friends of Anchor 2019, Grampian National Health Service-Endowments (Drs Sharma and Masannat), British Health Foundation PG/18/35/33786 to D. Dawson funded DG salary and BHF FS/RTF/20/30009 to D. Dawson funded AR salary.

### Disclosures

None.

### Supplemental Material

Supplemental MethodsFigure S1

References 42-43

## Supplementary Material


