# Supplementary material for: Impaired Cardiac and Skeletal Muscle Energetics Following Anthracycline Therapy for Breast Cancer
Source: Circ Cardiovasc Imaging. 2023 Oct 17;16(10):e015782. doi: 10.1161/CIRCIMAGING.123.015782 (PMC10581415; doi:10.1161/CIRCIMAGING.123.015782)
Supplement: Supplementary file 1 [file hci-16-e015782-s001.pdf]

Supplemental Material

Supplemental Methods:

Exclusion criteria for breast cancer patients were any previous cancer therapy, any significant cardiovascular disease such as valvular, coronary, or myocardial disease, diabetes or kidney disease or any contraindications to magnetic resonance imaging. A standard clinical examination, medical and medication history were performed in addition to routine blood tests undertaken for clinical indications.

For **^{31}P-Magnetic Resonance Cardiac and Skeletal Muscle Spectroscopy**, the participants were in a supine position. B_0 shimming was performed over the entire heart using a projection-based method. Following shimming and immediately prior to the ^{31}P acquisition the field homogeneity was manually assessed by measuring the linewidth from a ^{1}H point resolved spectroscopy voxel placed in the interventricular septum. ^{31}P- Magnetic Resonance Spectroscopy signal localisation was performed using 1D-Chemical Shift Imaging using adiabatic excitation (flip angle = 90°) with 24 phase encoding steps positioned parallel to the chest wall with phase-encoding step size 18.75 mm, 4 averages, 2048 sample points and a sampling bandwidth of 3 kHz. Saturation bands were placed over the chest wall and the liver to minimise spectral contamination. For the skeletal muscle acquisition, a similar 1D-Chemical Shift Imaging protocol was followed, using 20 phase-encoding steps positioned laterally across the thigh, with a step size of 18.75 mm, 8 averages, 2048 sample points and bandwidth 3 kHz. All ^{31}P-cardiac spectroscopy acquisitions were prospectively ECG-gated, triggered to mid-late diastole, with a typical repetition time of ≥10 seconds. Respiratory triggering was not found to improve spectral quality and so was not employed. All 31P acquisitions were performed using a 140 mm transmit/receive phosphorus coil (Philips Healthcare, Best, NL). All spectroscopy data were analysed in JMRUi 5.2 using the advanced method for accurate, robust, and efficient spectral fitting (AMARES) algorithm [42] using spectra extracted from phase-encoded planes containing the interventricular septum

and the vastus lateralis muscle for ^{31}P cardiac and skeletal muscle spectral acquisitions respectively, with the phase-encoding planes of interest manually identified based on localiser 4-chamber and short-axis images. Cramér-Rao standard deviations of all peaks were calculated, and only those <20% were accepted for inclusion in the analysis.

For **cardiac magnetic resonance imaging**, the imaging protocol comprised the following: 1) balanced steady state free precession cine imaging in long axes and a full ventricular short axis stack (echo time (TE) 1.49ms, repetition time (TR) 3ms, field of view (FOV) 250mm foot – head (FH) x 250mm anterior to posterior (AP) x 107mm right to left (RL), voxel size 1.8mm FH x 1.8mm AP, 45° flip angle, slice thickness/gap 7/3 mm with image acceleration using Philips compressed sense with 30 cardiac phases); 2) balanced gradient echo readout with a Modified Look-Locker Inversion recovery (MOLLI, 5[3]3) scheme native T1 mapping (TE 0.94ms, TR 2.0ms, field of view (FOV) 300mm FH x 300mm AP x 47mm RL, voxel size 2mm FH x 2mm AP, 20° flip angle, slice thickness/gap 10/8.5); 3) Gradient And Spin Echo readout T2 mapping (TE 0.75ms, TR 1.92ms, field of view (FOV) 300mm FH x 300mm AP x 48mm RL, voxel size 2mm FH x 2mm AP, 20° flip angle, slice thickness/gap 10/9); 4) early and late post gadolinium enhancement (0.1 mmol kg^{-1} gadolinium diethylenetriamine-pentacetate, Gadovist; Bayer, Leverkusen, Germany) using a spoiled gradient echo inversion recovery sequence (TE 3.0ms, TR 6.1ms, FOV 320mm FH x 356mm AP x 108mm RL, voxel size 1.8mm FH x 2.2mm AP, 25° flip angle, slice thickness/gap 8/2mm) with swapping of the phase-encoding direction to exclude artefact, matching the cine images and 5) post-contrast T1 enhanced balanced gradient echo readout with a MOLLI 5[3]3 scheme acquired at exactly 10 min after contrast administration (TE 0.94ms, TR 2ms, FOV 300mm FH x 300mm AP x 47mm RL, voxel size 2mm FH x 2mm AP, 20° flip angle, slice thickness/gap 10/8.5mm). Gadolinium was not administered to healthy volunteers. Endocardial and epicardial borders of the motion corrected parametric maps were traced manually and set 10% inwardly to avoid blood on the endocardial side and fat or

extracardiac structures on the epicardial side. Epicardial, endocardial and papillary muscle borders were automatically detected and then adjusted manually to ensure they were accurately traced. A minority of artefactual images without clear epicardial and endocardial borders were excluded. All parametric images were collected using the Philips implementation of a motion corrected elastic image registration technique [43]. The myocardial extracellular volume fraction was calculated for the whole left ventricle using apical, mid-cavity and basal segments according to the formula:

$$ECV = (1 - hematocrit)\frac{\left(\frac{1}{T1_{myo\ post}} - \frac{1}{T1_{myo\ pre}}\right)}{\left(\frac{1}{T1_{blood\ post}} - \frac{1}{T1_{blood\ pre}}\right)}$$

.

For **2D-echocardiography**, three cine loops in each of the standard recommended British Society of Echocardiography views (parasternal long-axis, short-axis, and apical four-, three-, and two-chamber views) were obtained at a frame rate of at least 85 Hz. Together with colour and continuous/pulsed wave doppler examination a full dataset was stored for offline analysis. Left ventricular ejection fraction was calculated using the Simpson’s biplane method. Global longitudinal strain (GLS) was obtained after manually adjusting the automatic detection of the epicardial and endocardial borders in each of the three long axis views.

For **cardiovascular biomarkers**, venous blood was clotted and serum was separated by centrifugation at 50 *g* for 10 minutes, frozen and stored at -80°C. Samples were batch analysed in the Biochemistry Laboratory for N-terminal pro B-type natriuretic peptide (NT-pro-BNP) and high sensitivity Troponin-I using an Abbott Alinity I Immunoassay System Analyser (Abbott, Illinois, U.S.A.).

For **skeletal muscle biopsies**, the skin and subcutaneous tissue overlying the medial aspect of the left vastus lateralis (approximately 15 cm above the patella) was anesthetized with 2% lidocaine.

For **Real-time quantitative PCR assessment of mitochondrial copy number,** skeletal muscle samples were homogenised using a QIAGEN® Tissue Lyser II. Optimal lysis and purification of high-quality total RNA was performed using QIAGEN® RNeasy Fibrous Tissue Mini Kit. Uniform amplification of all transcripts was achieved using the QuantiTect Whole Transcriptome Kit. A two-step qRT-PCR reaction was performed using Eurogentec Reverse Transcriptase Core kit and Takyon™ Probe 2X MasterMix dTTP.

For **transmission electron microscopy**, after fixation (4% PFA, 15% sPA in 100mM PB pH 7.4) of human biopsies following treatment with osmium tetroxide and uranyl acetate, samples were dehydrated, and embedded in Durcupan resin (Fluka, Switzerland). Ultrathin sections were prepared (Ultracut S) and adsorbed onto glow-discharged Formvar-carbon-coated copper grids. Images were recorded under a Zeiss LEO 910 electron microscope equipped with a TRS sharpeye CCD camera (TRS Systems). Analysis and quantitation were done using ImageSP software (TRS Systems).

For **confocal laser microscopy**, samples from breast cancer patients before and after chemotherapy and healthy volunteers were fixed in 0.1 M phosphate-buffered saline (PBS; ThermoFisher Scientific) containing 4% paraformaldehyde (PFA) and 15% saturated picric acid at 4°C overnight. Samples were dehydrated by ascending ethanol series, embedded in paraffin, and thin-sectioned (5-7 µm) using a microtome (RM 2235, Leica). Paraffin sections were rehydrated, blocked in peroxidase-containing buffer, and citrate-EGTA antigen recovery was performed. Slides were rinsed with PBS and blocked with 5% bovine serum albumin (BSA; Sigma-Aldrich) in the presence of 0.5% Triton X-100 for 60 min. Then, sections were incubated with primary antibodies Titin-Z, (Myomedix, Manheim, Germany) and ACTN2 (Sigma A7811) followed by fluorophore-conjugated secondary antibodies at 4°C overnight. Samples were embedded in Dako fluorescent mounting medium (Sigma F4680) to reduce

bleaching. Imaging was done with a confocal laser scanning microscope (Leica TCS SP8) using a 63× oil Plan-Apochromat objective.

Supplemental figures and figure legends:

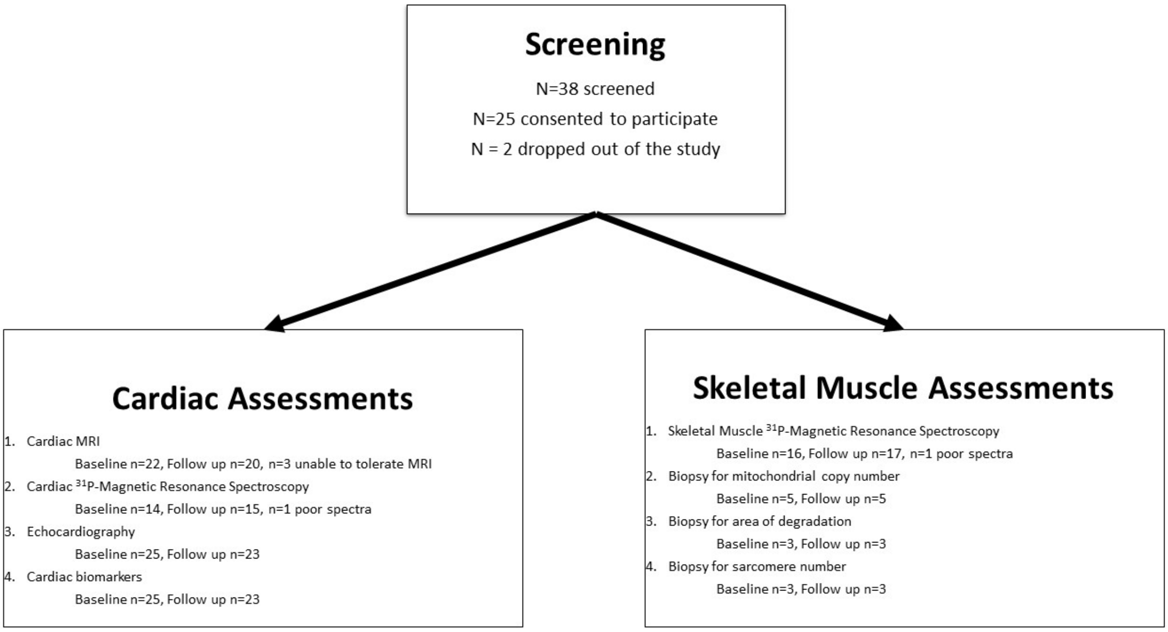

Figure S1 – flow diagram showing the number of participants screened, recruited and dropped out and the numbers of participants who underwent each investigation.
